# Server Consolidation Based on Culture Multiple-Ant-Colony Algorithm in Cloud Computing

**DOI:** 10.3390/s19122724

**Published:** 2019-06-17

**Authors:** Chunmiao Yuan, Xuemei Sun

**Affiliations:** School of Computer Science and Technology, Tianjin Polytechnic University, Tianjin 300387, China; seesea_sun@163.com

**Keywords:** cloud computing, data center, energy consumption, culture multiple-ant-colony algorithm, server consolidation

## Abstract

High-energy consumption in data centers has become a critical issue. The dynamic server consolidation has significant effects on saving energy of a data center. An effective way to consolidate virtual machines is to migrate virtual machines in real time so that some light load physical machines can be turned off or switched to low-power mode. The present challenge is to reduce the energy consumption of cloud data centers. In this paper, for the first time, a server consolidation algorithm based on the culture multiple-ant-colony algorithm was proposed for dynamic execution of virtual machine migration, thus reducing the energy consumption of cloud data centers. The server consolidation algorithm based on the culture multiple-ant-colony algorithm (CMACA) finds an approximate optimal solution through a specific target function. The simulation results show that the proposed algorithm not only reduces the energy consumption but also reduces the number of virtual machine migration.

## 1. Introduction

Cloud computing is one of the most important changes in the field of the computer industry recently. It develops rapidly and provides users with almost unlimited virtual computing, storage and network resources. Users only need to purchase the required resources from the cloud providers on an on-demand basis. In order to meet the growing needs of users, the size and energy consumption of cloud data centers are increasing [1], as noted in the report issued by the Department of Energy in America, A typical data center accounts for 1.5% of all energy consumption in the United States [2], and the demand for electricity is still growing at a rate of 12 percent. High-energy consumption not only transforms into high operating cost, but also leads to high carbon emissions. As a result, energy consumption in data centers has become a major concern.

In recent years, some attempts have been made to reduce the energy consumption of data centers [3,4]. One effective and commonly used method is server consolidation. Server consolidation is to reduce the energy cost of the data center by migration of the virtual machine to fewer servers, and then some servers are shut down or worked in a low power state according to the resource requirement of virtual machines. There are many existing methods of server consolidation. The main conception of these methods is to use active virtual machine migration to consolidate virtual machines periodically, so that some low-load physical machines can be released and then terminated. The main research of dynamic server consolidation is to decide when to reallocate virtual machines from overloaded physical machines [5,6], as this directly affects resource utilization. In previous studies [7], two static threshold methods were used to indicate the time of reallocation of virtual machines. This method maintains the CPU efficiency of each physical machine between their thresholds, but it has no obvious effects on setting the static threshold of the dynamic working environment.

In this paper, for the first time ever, a dynamic server consolidation algorithm based on the culture multiple ant-colony algorithm (CMACA) is proposed to optimize the placement of virtual machines. CMACA uses artificial ants to consolidate virtual machines. The idea is to reduce the number of active physical machines according to the current resource requirements. These ants establish migration plans in parallel with specific objective functions. In order to evaluate the feasibility of the algorithm, we simulate the trajectory of real workload by CloudSim [8]. Simulation results show that CMACA not only reduces the energy consumption, but also reduces the number of virtual machine migration frequency. In addition, we use the distributed system framework [9] to perform dynamic server consolidation, which can improve the resource utilization and reduce energy consumption of the physical machines.

## 2. Relative Research 

At present, dynamic consolidation algorithms are based on particle swarm optimization, the greedy algorithm, ant colony algorithm and other heuristic algorithms. In the heuristic algorithm based on the greedy algorithm [10,11,12], reference [10] used the greedy algorithm to determine the orders in which loaded virtual machines migrate to light-loaded physical machines. Reference [11] used a minimum of balancing algorithm to maintain the loading balance among physical machines. In reference [12], the problem of server consolidation was considered as a random packing problem, and the dynamic bandwidth requirements of the virtual machine were consolidated by the online packing algorithm. The traditional heuristic algorithm based on the greedy algorithm can optimize the allocation of virtual machine, but the search strategy of single node is usually applied, so it is easy to fall into local optimum, and cannot achieve the optimal result of consolidation, which still needs further improvement.

In addition, the heuristic algorithm based on the ant colony algorithm, such as the ant system (AS), maximum ant system, (MMAS) and ant colony system (ACS) [13,14,15], an approximate optimal solution can be found through the ant colony system to complete server consolidation. The existing resource allocation and server consolidation algorithms based on the ant colony system include the following as [9,16,17,18,19]. In reference [16], the ant colony system is used to solve the problem of nonlinear resource allocation, the purpose of which is to find out some tasks of optimal allocation of a finite number of resources in order to optimize its nonlinear objective functions. Reference [17] applies the maximum and minimum ant colony system to the server consolidation problem in cloud computing. Reference [18] is the application of ant colony systems to consolidate multiple Web applications in a cloud-based environment of shared server machines.

In paper [19], an algebra vector algorithm of virtual machine consolidation (AVVMC) is proposed by combining ant colony systems with the capture techniques of server resource utilization based on vector algebra [20]. The main idea of this algorithm is to set the upper and lower utilization thresholds and maintain the total CPU utilization of one node between them. When the utilization rate exceeds the threshold, there is a load balancing redistribution of the virtual machines. When the utilization is below the lower threshold, the virtual machines are consolidated to make the redistribution. In reference [9], server consolidation is regarded as a multi-object combinatorial optimization problem. A server consolidation algorithm based on the ant colony system (ACS-VMC) is proposed, and an approximate optimal solution is obtained by using an adaptive online heuristic optimization algorithm [21]. This algorithm is one of the best methods to solve the problem of server consolidation using the ant colony algorithm. However, in the literature above, the single colony is taken as the direction for consideration. Compared with the multiple-ant-colony algorithm (MACA) [22], there is a deficiency in the diversity of solutions, similar to the greedy algorithm, which is prone to cause local optimal problems in server consolidation, thus imposing a certain effect on reducing energy consumption and migration times of the virtual machine.

In this paper, a virtual machine consolidation algorithm based on the culture multiple-ant-colony algorithm (CMACA) was designed. This algorithm improves the deficiency of the greedy algorithm in the process of virtual machine placement and avoids the difficulty of particle swarm optimization in dealing with discrete problems. The unicity of the ant colony algorithm is improved to get a better algorithm of server consolidation. In this algorithm, each artificial ant consolidates the virtual machine of the low utilization physical machine into the more active physical machine according to the current resource situation, so as to reduce the number of working physical machines. It not only reduces the energy consumption of the cloud data center, but also increases the utilization rate of the physical machine resource in the cloud computing data center.

The main contribution of this paper was to introduce CMACA into the server consolidation problem for the first time. CMACA was proposed and compared with the ACS-VMC mentioned above. The simulation results showed that CMACA outperforms ACS-VMC in reducing energy consumption and virtual machine migration times.

## 3. System Modeling

### 3.1. Model of Server Consolidation System

There are *m* different physical machines (PMs) in a cloud data center, and the resource capacity of these machines is different. Usually, physical machines can be divided into four categories: Normal physical machine (*P_normal_)*, overloaded physical machines (*P_over_*), predicted overloading physical machine (${\hat{P}}_{\mathrm{over}}$) and light-loaded physical machine (*P_under_*); the basis of this classification is as follows:

1. If the current CPU utilization exceeds the capacity of the physical machine in the environment, the physical machine can be defined as an overloaded physical machine (*P_over_*).
2. If the predicted CPU utilization is greater than the capacity available for CPU, the machine is considered as a predictive overloaded physical machine. LIRCUP [23] based on a linear regression is used to predict the CPU utilization of physical machines in the short term.
3. If the current CPU utilization value is lower than the total CPU utilization threshold, the physical machine is a light-loaded physical machine.
4. All the other operating physical machines are defined as standard physical machines.

Each physical machine includes a multiple-core CPU, whose performance can be defined by the (MIPS) of the million-level machine-language instructions processed per second. At any given time, a cloud data center often serves multiple users at the same time. Users submit their requests to physical machines, which then assign tasks to *N* virtual machines. The length of each request is divided by thousands of instruction (MI).

**Figure 1:**
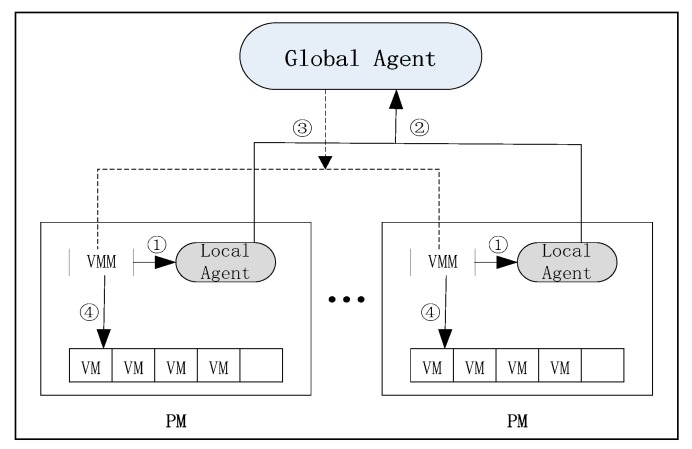
Model of the server consolidation system.

Figure 1 depicts the system model [24], which consists of two types of agents: Local agents and global agents. There is a local agent in each physical machine to determine the state of the physical machine by observing the most recent resource utilization of the physical machines. The global agent acts as a supervisor and optimizes the placement of virtual machines (VMs) by CMACA. The model works as follows:

1. The local agent (LA) monitors the utilization of CPU and classifies physical machines.
2. The global agent (GA) collects the state of each physical machine, and uses CMACA to establish a globally optimal migration plan, which will be described in detail in the part of algorithm description.
3. The global proxy sends a command to virtual machine management (VMM) to perform the migration consolidation task, which determines which virtual machines need to be migrated to which destination machine.
4. When VMM receives instructions from GA, it begins to execute real virtual machine migration plans.

In this paper, CMACA firstly realized the server consolidation of the multiple-ant-colony algorithm (MACA), and then on the basis of this research, MACA was transplanted into the framework of cultural algorithm to form CMACA.

### 3.2. Definition of Sever Dynamic Integration Model

Cloud data centers consume a large amount of energy while providing reliable services. In order to reduce energy waste and improve energy efficiency, multiple virtual machines can be integrated into a physical machine to improve the resource utilization of the physical machine and reduce the number of physical machines turning on, so as to achieve the purpose of energy saving. To ensure quality of service, the total demand for CPU and memory for all virtual machines running on a physical machine cannot exceed the m[aximum resource supply capacity of the physical machine. Due to the continuous change of workloads, it is necessary to use virtual machine migration technology to migrate VMs dynamically in order to improve the resource utilization of physical machines and guarantee service performance. The model of dynamic integration of virtual machines for system energy consumption optimization [25] can be depicted in (1) as the following single objective optimization model:

Objective function:

(1) $$\min\sum_{j=1}^{m}p(PM_{j})\cdot y_{j}=\min\sum_{j=1}^{m}(p_{j}^{idle}+\mu_{j}^{cpu}(P_{j}^{\max}-P_{j}^{idle}))\cdot y_{j}.$$

s.t. $\sum_{i=1}^{n}V_{i}^{cpu}\cdot x_{i,j}\leq PM_{j}^{cpu}\forall j\in \{1,2,\ldots ,m\}\;$, ①

$\;\sum_{i=1}^{n}V_{i}^{men}\cdot x_{i,j}\leq PM_{j}^{mem}\forall j\in \{1,2,\ldots ,m\}\;$, ②

$\;\sum_{i=1}^{n}x_{i,j}=1\;\forall i\in \{1,2,\ldots ,n\}\;$, ③

$\;x_{i,j}=\{0,1\}\;\forall i\in \{1,2,\ldots ,n\}\;,\forall j\in \{1,2,\ldots ,m\}\;$, ④

$\;y_{j}=1,\;\mathrm{if}\sum_{i=1}^{n}x_{i,j}>0;\;y_{j}=1,\;\mathrm{if}\sum_{i=1}^{n}x_{i,j}=0\;\forall j\in \{1,2,\ldots ,m\}\;$. ⑤

Equation (1) is the objective function to minimize the total energy consumption of cloud data centers. Constraint ① represents all virtual machines placed on the physical machine *PM_j_*, and the sum of the power required of CPU processing cannot exceed the maximum CPU processing power of that physical machine. Constraint ② means that the requested memory size of all virtual machine placed on physical machine *PM_j_* cannot exceed the memory size of the physical machine. Constraint ③ means that each virtual machine must be placed on a physical host. Constraint ④ defines *x_i,j_* as a Boolean variable. When the virtual machine *i* is placed on the physical machine *j*, *x_i,j_* is set to 1, otherwise *x_i,j_* is set to 0. In constraint ⑤, *y_j_* is also defined as a Boolean variable. When the physical machine *j* turns on, *y_j_* is set to 1, and when it shutdowns, *y_j_* is set to 0.

## 4. Server Consolidation Based on Culture Multiple-Ant-Colony Algorithm

### 4.1. Algorithm Framework

Cultural algorithm is a kind of two-level evolutionary algorithm. We put different evolutionary algorithms into the two spaces of the cultural algorithm, then we can realize the evolutionary solution of the two spaces at different speeds. Among them, the multiple-ant-colony algorithm is put into its population space, the multiple-ant-colony algorithm is used to simulate the process of the natural optimal solution, and then the colony optimal solution is transferred to the belief space, and the optimal solution is mutated twice through the evolution rules of the belief space, thus achieving the goal of finding the best value quickly.

**Figure 2:**
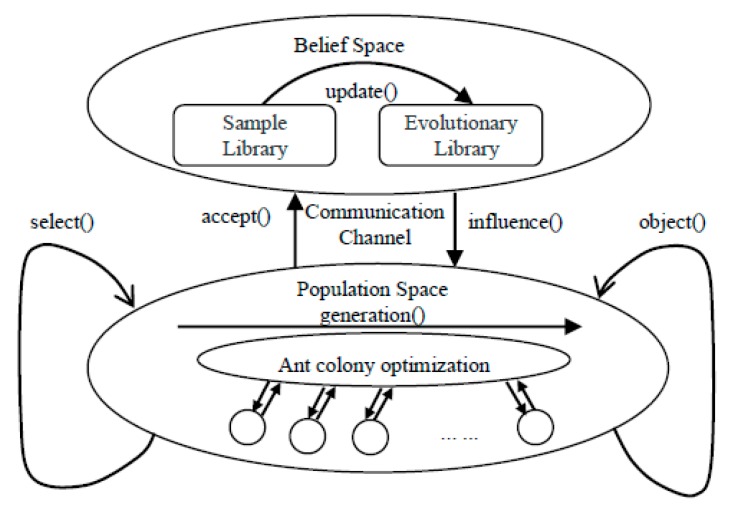
Framework of the culture multiple-ant-colony algorithm (CMACA).

As is shown in Figure 2, in the cultural algorithm, the MACA is used in the population space, the *select*() method is used to find the optimal solution of the colony in the population space, and the optimal solution of the colony is transferred to the belief space sample library by the *accept*() method. The *update*() function in belief space is used to evolve the optimal solution of the colony. If the solution is better than the optimal solution in the belief space evolution library, then the evolutionary library is to be updated, and the ant offspring in the population space is to be induced to evolve by using the *influence* () method. In the population space, some individuals are selected as the next generation parent by the *object* () method, that is, the ant colony that obtains the global optimal solution is the parent generation, and then each colony uses the *generate* () function to generate the next generation multiple-ant-colony until the optimal solution is found.

### 4.2. Flow of the Algorithm

**Figure 3:**
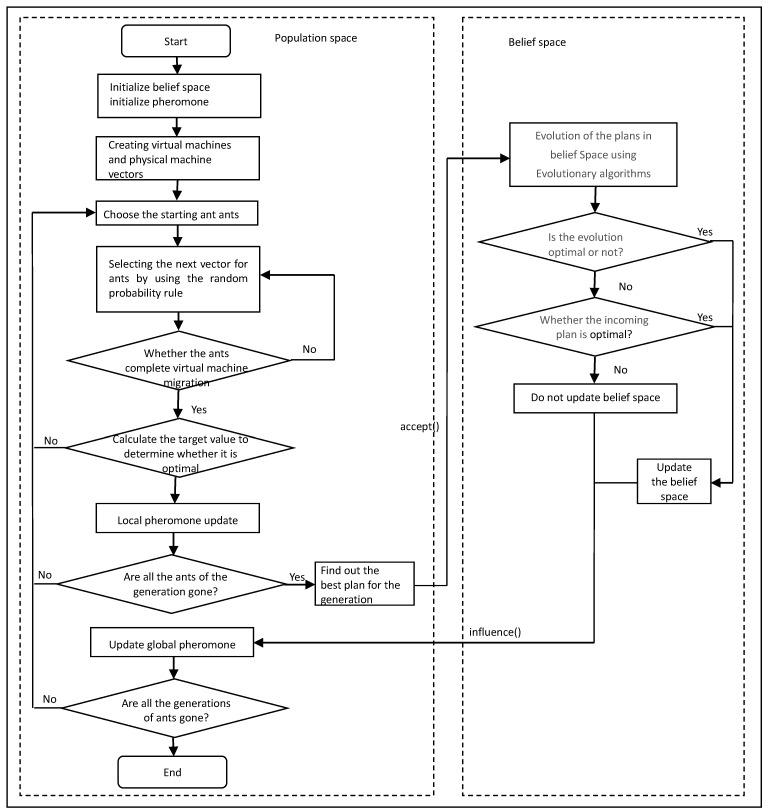
Flow chart of CMACA.

The flow chart of CMACA is shown in Figure 3. First, initialize the belief space and pheromone matrix to create vectors of virtual machines and physical machines; then set the starting point for each ant and select the next vector for the ant by using the random probability rule. After each ant completes the whole migration plan, the target value of this migration is calculated to determine whether the colony is optimal. If so, the local pheromone is updated, and if not, the plan is abandoned. When the ant generation has completed the migration plan, the optimal migration plan is passed into the belief space through the *accept ()* function, and it evolves in the belief space to determine whether there is a better plan. If there is, the belief space is updated, and the *influence ()* function is used to guide the offspring ants in the population space to find their ways. If not, the belief space is not updated, and the ants return to the population space.

### 4.3. Population Space Design

Suppose one or more virtual machines may be placed on each physical machine p∈P. In the plan of virtual machine migration, each physical machine is a potential source physical machine, because there may already have been virtual machines hosted on this physical machine. The same virtual machine can be migrated to all other physical machines, so all the other physical machines are the potential physical machines. Firstly we created a set of tuples T, each of which contained three elements: The source physical machine *P_so_*, the virtual machine *v* to be migrated, and the destination physical machine *P_de._* The description is as follows (2):

(2) $$t=(p_{so},v,p_{de})$$

The output of the server consolidation algorithm is a migration plan, and the result is that all virtual machines can be satisfied with the least active physical machines without any degradation of performance. The objective function of this algorithm is described as (3):

(3) $$f(M)=|P_{s}{|}^{\gamma }+\frac{1}{\left|M\right|}$$

where *M* is a migration plan, *P_s_* is a set of physical machines that will be turned off to sleeping mode when *M* is executed. The parameter γ determines the importance of the |*P_s_*| relative to the |M|. Since the final goal of the dynamic server consolidation algorithm is to reduce the number of active physical machines, the objective function is defined according to the number of physical machines in the sleeping mode.

At the end of the algorithm, when the selected migration plan is executed, the number of active physical machines is constrained by migrating the virtual machine to the active physical machine. A physical machine is turned off to sleeping mode only if it is not possible for a virtual machine to migrate from the physical machine to another one and the physical machine is not hosting a virtual machine. It is as follows (4):

(4) $$P_{s}=\{\forall p\in P|V_{p}=\emptyset \}$$

where there is a collection of virtual machines *V_p_* on a physical machine *P*.

In this paper, pheromones are placed in the tuples defined in (1). Each ant *nA* uses the transition rule at the random state to select the next tuple to find its way. In the ant colony system, the rule of state transition is called pseudo-random proportional rule. By this rule, an ant *k* finds its path by selecting tuples *s* in the following form, which is described as the expression (5):

(5) $$S={\{}_{S,q>q_{0}}^{\arg\max u\in T_{K}\{[\tau_{u}]\cdot {\left[\eta_{u}\right]}^{\beta }\},q\leq q_{0}}$$

In the function, τ is the number of pheromones, η is the heuristic values of specific tuples and β is the element that affects the number of pheromones. $T_{k}\in T$ is the untraversed tuple that ant *k* tries to travel in tuple *T.* q∈[0,1] in the tuple *T* is a random variable with uniform distribution, and the parameter *S* is a random variable selected by the formula of probability distribution given by formula (6). The probability definition of an ant *k* random selection *P_s_* for the next path finding is as (6):

(6) $$p_{s}={\{}_{0,otherwise}^{\frac{[\tau_{s}].{\left[\eta_{s}\right]}^{\beta }}{\sum_{u\in T_{k}}[\tau_{u}].{\left[\eta_{u}\right]}^{\beta }},s\in T_{k}}$$

The heuristic value of Tuple *s*, $\eta_{s}$ is defined such as (7):

(7) $$\eta_{s}=\left\{ \begin{array}{l} (\left|C_{P_{de}}-(U_{P_{de}}+U_{v})\right|){}^{-1},U_{P_{de}}+U_{v}\leq C_{P_{de}}\\ 0,otherwise \end{array}\right..$$

Among them, $C_{P_{de}}$ is the total capacity value of the target physical machine $P_{de}$, $U_{P_{de}}$ is the used capacity value of the target physical machine and $U_{v}$ is the capacity value of the virtual machine in the tuple s. The heuristic value is based on the multiplicative inverse elements of scalar values between $C_{P_{de}}$ and $U_{P_{de}}$ + $U_{v}$. This allows the virtual machine migration to reduce the number of low-utilization physical machines. Furthermore, the constraints $U_{P_{de}}$ + $U_{v}$ <= $C_{P_{de}}$ prevent the target physical machines from becoming an overloaded ones.

In addition to the transition rules at the random state, the global and local pheromone update rules are utilized after all the ants have completed their migration plans. The global pheromone update rules are defined as (8):

(8) $$\tau_{s}=(1-\alpha ).\tau_{s}+\alpha .\Delta_{\tau_{s}}^{+}.$$

$\Delta_{\tau_{s}}^{+}{}_{}$ is the number of additional pheromones, which is only given to tuples that belong to the best global migration plan. $\Delta_{\tau_{s}}^{+}{}_{}$ is defined as follows (9):

(9) $$\Delta_{\tau_{s}}^{+}={\{}_{0,otherwise}^{f(M^{+}),s\in M^{+}}.$$

α∈(0,1] is the pheromone decay parameter, *M^+^* is the best global migration plan. 

When an ant makes a migration plan through a tuple at the same time, it applies the local pheromone trace rule, which is defined as (10):

(10) $$\tau_{s}=(1-\rho ).\tau_{s}+\rho .\tau_{0}.$$

Among them, ρ∈(0,1] is similar to and is the initial pheromone standard of α and $\tau_{0}$, $\tau_{0}$ is defined as (11):

(11) $$\tau_{0}={\left(|M|.|P|\right)}^{-1}.$$

In this paper, the K-neighbor node heuristic algorithm was used to track the data set to estimate the optimal *|M|*. The data set had *M* samples, each sample had three inputs *(x_i1_,x_i2_,x_i3_)* and one output *y_i_*, that was, *x_i_ = (x_i1_, x_i2_, x_i3_, y_i_)*. The aim of this operation was to find out the relationship between the input and output, so we chose the number of light-loaded physical machines, overloaded physical machines and the virtual machines as the input, and the size of the migration plan as the output.

When a colony completes the search, the information entropy of each ant is calculated according to the *P_s_* value of each ant, such as (12):

(12) $$s=-k\sum_{i=1}^{n}p_{i}\ln p_{i}.$$

*N* represents the number of ants in the colony, and the information entropy of the colony obtained will be used as the basis of the colony selected for the exchange of pheromones among the colony.

In the algorithm, after intervals with a certain time, the colony will communicate. The communication group is determined according to the information entropy of each group. Suppose group *i* select group *j* as the object of information exchange, then *j* can be determined by (13):

(13) $$j=\underset{1\leq j\leq h}{\arg\max}(|s_{i}-s_{j}|).$$

*S_i_* and *S_j_* are the information entropy of groups *i* and *j* at present. Therefore, the group with larger information entropy will choose the group with smaller information entropy to exchange information, so that the group pheromone distribution with small information entropy can balance their pheromone distribution through the communication between the groups with high information entropy. The communication of groups with the same information entropy and small information entropy can concentrate on their own pheromone distributions.

(14) $$\tau_{uv}^{i}=\tau_{uv}^{i}+\lambda \Delta \tau_{uv}^{i}.$$

In (14), $\lambda =S_{i}-S_{j}$, $\lambda_{\min}$<λ<$\lambda_{\max}$, $\lambda_{\min}$ and $\lambda_{\max}$ are the constants, which stands for the minimum and maximum values of the renewal coefficient. $\Delta \tau_{{}_{uv}}^{i}$ is the pheromone of the sub-colony *j* on the path (*UV*). 

The time interval of colony exchange is not fixed, but is determined by the information entropy of all colony, that is to say, it changes with the convergence of all colonies. *Gap* for exchange meets (15):

(15) $$gap=k_{1}\cdot e^{\frac{\sum_{i=1}^{h}s_{i}}{h}}.$$

Among them, *k*_1_ is the constant, *h* is the number of sub-colony and *S_i_* is the information entropy of the *i* sub-colony.

### 4.4. Maintenance of the Belief Space

In the belief space, according to the availability of the physical machine, the unmigrated vector *V_k_,* is traversed to see if the virtual machine migration can be carried out in order to achieve a better result. That is, when $v\in V_{k}$*,* if the vacancy rate of the source physical machine is greater than the resource occupancy of the virtual machines, and the source physical machine has no other virtual machines, then the vector *v* is added to the migration plan, and by this step, all the *V_k_* are traversed until the end of the traversal. At this time, calculate and decide whether the target value is superior to the target value of the incoming belief space, and whether it is superior to the previous belief space optimal value, if the value is superior, the belief space evolution library needs to be updated.

### 4.5. Cmaca Pseudo-code Description

The pseudo-codes of CMACA (Algorithm 1) are described as follows.

**Algorithm 1.** CMACA Pseudo-Code1:   Initialize ant[m][n],bestM[m],target,bestTarget[m]; 2:   Initialize belief Space; 3:   Initialize tao[[][];// Initialize pheromones matrix 4:   createV();// Create vector 5:   For run∈[i,runtime] do // Number of iterations 6:        For m∈[1,groupNum] do // Number of ant colony 7:               For n∈[1,everyGroupNum] do // Number of ants in one colony 8:                     For v∈V do 9:                           Create random variable q between 0 and 1; 10:                         If q>q_0_ then 11:                               Calculate probability P by formula (5); 12:                         End if 13:                         ant[m][n].selectNextV();// select next vector 14:                         Partial update; 15:                         If ant[m][n] completed then 16:                               Target=ant[m][n].calTarget();// Calculate target value 17:                               If target>bestTarget[m] then 18:                               bestM[m]=ant[m][n].M; 19:                                     bestTarget[m]=target; 20:                                     updateTao();//Update pheromones 21:                         End if 22:             End for 23:             Accept();// Enter the belief space 24:             Update();// Evolve of incoming migration plans 25:             If updateTarget>culTarget&&updateTarget>bestTarget[m] then         // Evolution success 26:                   culM=updateM; 27:                   culTarget=updateTarget; 28:             else if bestTarget[m]>culTarget then 29:                         culM=bestM[m]; 30:                         culTarget=bestTarget[m]; 31:                   End if 32:             End if 33:             Influence();//Update pheromones 34:       End for 35: End for

## 5. Simulation Experiment and Analysis

We selected simulation software, CloudSim, to evaluate the performance of the algorithm, and a data center covering many different physical computers is simulated experimentally. Two server configurations in CloudSim were chosen: HP ProLiant ML110 G4 (Intel Xeon 3040, two cores × 1860 MHz, 4 GB), HP ProLiant ML110 G5 (Intel Xeon3075, two cores × 2660 MHz, 4 GB).) The specific parameters of these hosts are listed in Table 1. Characteristics of the VMs are depicted in Table 2. There are four kinds of VMs in Table 2, that is the large instance and small instance [26]. The environment was sufficient for evaluating the hardware configuration required for resource management methods based on multiple-core CPU architecture.

It is necessary and important to do experiments using real workload data. In our experiment, we used the workload derived from a CoMon project. The function of CoMon is to monitor infrastructure for PlanetLab [27]. Table 3 depicts the characteristics of the data [13].

The simulation was divided into three parts. Firstly, we compared our algorithm with IQRMC[13], LRMMT[13] and THRMU[13] algorithms of CloudSim under different workloads. Secondly, we compared our algorithm with ACS-VMC. Lastly, we simulated a datacenter with large amount hosts. To evaluate the effectiveness of the proposed method, we analyzed and evaluated three indicators: Energy consumption, the number of virtual machine migrations and average SLA(Service Level Agreement) violation.

### 5.1. Comparation under Different Workloads

**Figure 4:**
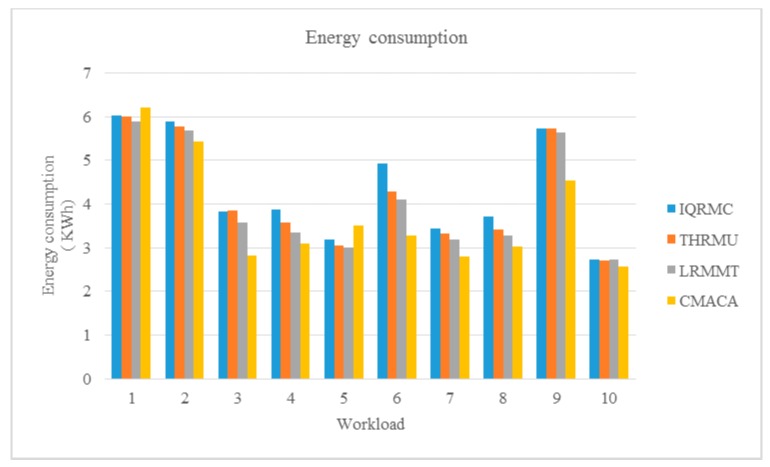
Comparison of energy consumption under different workloads.

**Figure 5:**
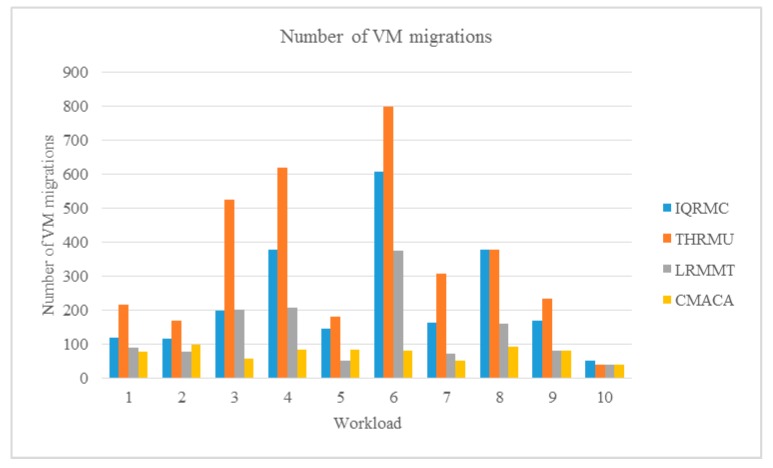
Comparison of the number of VM migrations under different workloads.

**Figure 6:**
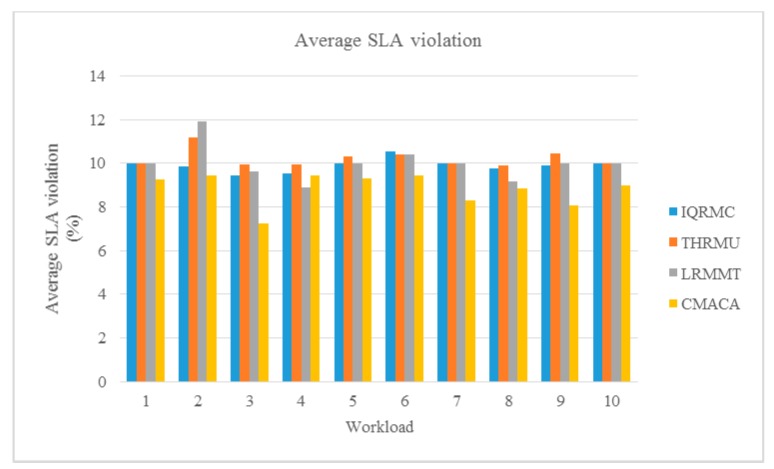
Comparison of the number of average SLA violation under different workloads.

We compared our algorithm with IQRMC, LRMMT and THRMU algorithms of CloudSim ten times. We simulated a data center, which included 21 physical hosts and 30 VM hosts and used a different workload from No.1 to No.10 as listed in Table 3. The experiment results are showed in Figure 4, Figure 5 and Figure 6.

The workload of the application programs should be taken into account while calculating the total energy consumption of the physical resources in a data center. It mainly considers its CPU efficiency, memory usage, hard disk occupation and network card usage when calculating the energy of a single physical computer. Literature [28] studies show that the power consumption of the memory disk generally does not change with the change of load, while the power consumption of CPU will change with the change of load. The CPU utilization of a physical machine is usually used to represent the resource utilization of the physical machine. In the experiment, LiRCUP based on linear regression was used to predict the CPU utilization of physical computers in the short term.

Dynamic server consolidation is a resource-consuming computing process, which can increase the CPU resource consumption of the source physical hosts and the bandwidth resource consumption between the source physical hosts and the destination hosts, pause the service on the migration virtual machine and increase the time of the total consolidation migration. Therefore, one of the goals of this algorithm was to minimize the number of migrations. The migration time of a virtual machine in CloudSim was similar to the migration time that memory was allocated to the virtual machine between the network bandwidth link of source machine and destination physical machine. The network link of 1Gbps was used in the simulation experiment.

Figure 4 shows the energy consumption of four algorithms under 10 workloads. In our algorithm, we used the object function and limited conditions as depicted in (1) and (7), which allowed the virtual machine migration to reduce the number of low-utilization physical machines and the constraints prevented the target physical machines from becoming overloaded ones. Therefore, we could see the experiment data showed that our algorithm had less energy consumption of the server consolidation.

We adopted the double evolutionary algorithm of the multi-ant colony and cultural algorithm in our algorithm in which an approximate optimal solution was found through a specific objective function. The multi-ant colony algorithm was used to simulate the process of seeking the optimal solution in reality, and then the optimal solution of population was transmitted to belief space. Through the evolutionary rules of belief space, the optimal solution was quadratically mutated to achieve the purpose of finding the optimal solution quickly. Therefore it could be seen that the number of migrations was reduced compared with other algorithms in virtual machine migration from the experimental results as shown in Figure 5.

SLA violations are an important factor for any VMs deployment, which represents that the SLA violation time accounts for the total time of the active host. The SLA violation time means the CPU utilization of the active host has reached 100% during the time. In CloudSim, it calculates the total time of all active hosts violating SLA and the total time of all active hosts, and then takes the ratio of them to get the ratio of SLA violating. From Figure 6, we could see that in our algorithm, the number of virtual machine migration was reduced, while the SLA value kept basically the same as other algorithms.

### 5.2. Comparison with ACS-VMC

In this part, the CMACA were compared with ACS-VMC. We simulated a data center that included 10 physical hosts and 20 VMs as described in Table 1 and Table 2. We still used different workloads from No.1 to No.10 as listed in Table 3. In order to better demonstrate the reliability of the simulation results, we performed with the same simulation environment and compared the energy consumption and number of migrations.

**Figure 7:**
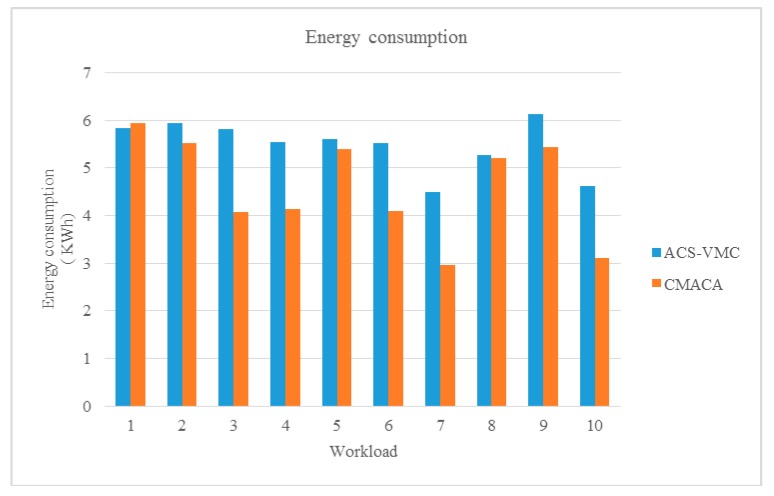
Comparison of energy consumption with the ant colony system (ACS).

In CMACA, we put the MACA into the population space of the culture algorithm, and the optimum value of each generation ant in the population space was passed through the function into the belief space of the cultural algorithm. The MACA was evolved by evolutionary algorithms in belief space in order to reduce the energy consumption of cloud data centers by making more physical machines to shutdown with the least number of migrations. As a result, it can be seen that the energy consumption of CMACA algorithm is less than that of ACS-VMC algorithm. The average energy consumption of the ACS-VMC algorithm was about 5.5 KWh, but comparatively, the average energy consumption of the CMACA algorithm was only less than 4.6 KWh, and it was relatively stable. From Figure 7, we could see that the algorithm of CMACA was much better than ACS-VMC in terms of energy consumption of the server consolidation.

**Figure 8:**
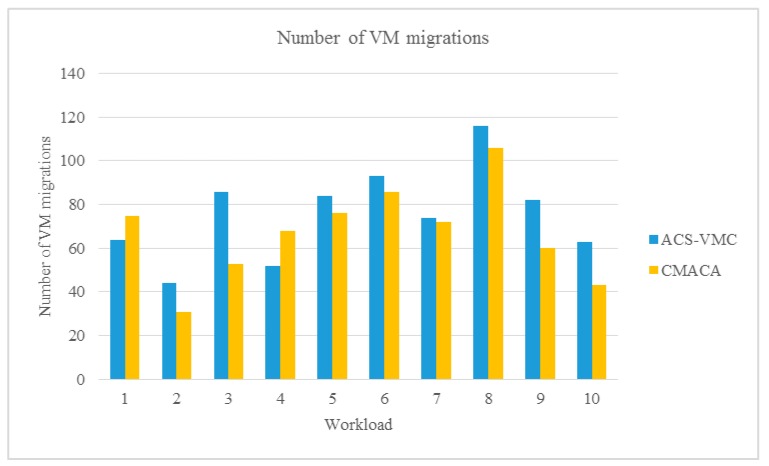
Comparison of the number of VM migrations with ACS.

As is shown in Figure 8, the number of virtual machine migrations of the CMACA algorithm hovered around an average of 67 times in ten experiments, and the ACS-VMC algorithm was about an average of 76 times. From the experimental result, we could see the number of virtual machine migration of CMACA algorithm was less than that of ACS-VMC algorithm after being evolved by cultural algorithms. The results showed that the proposed algorithm had significant advantages in reducing the number of virtual machine migration while saving energy.

### 5.3. Experiment Under Large Amount Servers 

Last, we simulated a data center that included 1000 physical hosts and 100 VMs and we compared the CMACA with IQRMC, LRMMT and THRMU algorithms under this experiment environment. Workload of date “12/April/2011” as listed in Table 3 was selected in this simulation. We performed operations ten times and calculated the average number of VM migrations and average energy consumption as shown in Table 4. When the host’s number was large, we still could see our algorithm was better than others.

From this experiment, we found when the workload was small and the host was large, the running time of the algorithm would increase greatly. Meanwhile, the number of migrations and energy consumption would corresponding grow. Therefore, the quantitative relationship between tasks and hosts should be considered carefully when allocating virtual machines.

## 6. Conclusions

In this paper, a server consolidation algorithm based on the culture multiple-ant -colony algorithm was proposed for the first time. Through simulation analysis, we could conclude that CMACA was much better than the algorithm based on the ant colony system in terms of energy consumption and migration times. However, as for the number of sleeping physical machines, there was no significant difference between the two. Therefore, in subsequent studies, emphasis could be placed on increasing the number of sleeping physical machines. In addition, we intend to study other heuristic algorithms on server consolidation to further improve the algorithm. Meanwhile, it was planned to use heterogeneous MACA as an extended virtual machine manager to evaluate the performance of the server consolidation algorithm in a real cloud environment on a public clouding platform.

## Figures and Tables

**Table 1:** Configuration of servers.

Servers	MIPS	PES	RAM/GB	BW/(Gbit/s)	STORAGE/GB
Host1	1860	2	4	1	1
Host2	2660	2	4	1	1

**Table 2:** Four kinds of virtual machine (VM) types.

VM	MIPS	PES	RAM/MB	BW/(Mbit/s)	STORAGE/GB
VM1	2500	1	870	100	1
VM2	2000	1	1740	100	1
VM3	1000	1	1740	100	2.5
VM4	500	1	613	100	2.5

**Table 3:** Workload details.

No	Date	Number of VMs	Mean	Quartile 1	Quartile 3.
1	3 March 2011	1052	12.31%	2%	15%
2	6 March 2011	898	11.44%	2%	13%
3	9 March 2011	1061	10.70%	2%	13%
4	22 March 2011	1516	9.26%	2%	12%
5	25 March 2011	1078	10.56%	2%	14%
6	3 April 2011	1463	12.39%	2%	17%
7	9 April 2011	1358	11.12%	2%	15%
8	11 April 2011	1233	11.56%	2%	16%
9	12 April 2011	1054	11.54%	2%	16%
10	20 April 2011	1033	10.43%	2%	12%

**Table 4:** Result of the experiment when servers number = 1000.

Algorithms	Number of VM Migrations	Energy Consumption (kwh)
IQRMC	1329	13.13
LRMMT	1230	12.68
THRMU	3424	13.21
CMACA	1204	11.24
